# Stabilization of molten salt materials using metal chlorides for solar thermal storage

**DOI:** 10.1038/s41598-018-26537-8

**Published:** 2018-05-29

**Authors:** T. O. Dunlop, D. J. Jarvis, W. E. Voice, J. H. Sullivan

**Affiliations:** 1 College of Engineering, Swansea University, Bay Campus, Fabian Way, Swansea, SA1 8EN UK; 2 Gervaux Ltd, 79 Davies Road, West Bridgford, Nottingham, NG2 5JB UK

## Abstract

The effect of a variety of metal-chlorides additions on the melting behavior and thermal stability of commercially available salts was investigated. Ternary salts comprised of KNO_3,_ NaNO_2,_ and NaNO_3_ were produced with additions of a variety of chlorides (KCl, LiCl, CaCl_2_, ZnCl_2_, NaCl and MgCl_2_). Thermogravimetric analysis and weight loss experiments showed that the quaternary salt containing a 5 wt% addition of LiCl and KCl led to an increase in short term thermal stability compared to the ternary control salts. These additions allowed the salts to remain stable up to a temperature of 630 °C. Long term weight loss experiments showed an upper stability increase of 50 °C. A 5 wt% LiCl addition resulted in a weight loss of only 25% after 30 hours in comparison to a 61% loss for control ternary salts. Calorimetry showed that LiCl additions allow partial melting at 80 °C, in comparison to the 142 °C of ternary salts. This drop in melting point, combined with increased stability, provided a molten working range increase of almost 100 °C in total, in comparison to the control ternary salts. XRD analysis showed the oxidation effect of decomposing salts and the additional phase created with LiCl additions to allow melting point changes to occur.

## Introduction

The potential of solar energy as a sustainable and affordable power source has resulted in a great deal of worldwide interest. Technological developments have progressed at great speed over the past decade, particularly within the field of solar thermal energy and photovoltaics^1^. Solar thermal plants can be used to concentrate the Sun’s energy and convert it into electricity using molten salt mixtures and water steam generators. Utilization of relatively inexpensive molten salts also allows a period of heat storage, enabling the release of energy overnight.

Within Concentrated Solar Power (CSP) applications molten salts can be used as Heat Transfer Fluids (HTF) and/or as for Thermal Energy Storage (TES). TES technology is key for the efficiencies of solar thermal plants, HTF’s allow electricity to be generated when there is no sunlight present. Molten salts can be used to improve plant efficiency and reduce cost by allowing more efficient device operation. For maximum efficiency, it is desired that the salts should have a low melting point and high temperature stability. The wider the working range the more effective the salt will be, working range is the operating temperature between the melting point and high temperature decomposition of the material. Two traditionally popular salt mixtures have been Solar Salt, a binary mixture of sodium nitrate (NaNO_3_) and potassium nitrate (KNO_3_), and HITEC (53 wt% KNO_3_ – 40 wt% Sodium Nitrite (NaNO_2_) – 7 wt% NaNO_3_)^2,3^. The addition of sodium nitrite to the traditional binary mixture has reduced the melting temperature from 220 °C of the Solar Salt to 142 °C. HITECs upper operating temperatures have been reduced from the Solar Salts 600 °C to 454 °C for long term (and 538 °C for short term behaviour), giving a long term working range of 312 °C. Additions to this ternary mixture have been used to improve the stability somewhat, but little stability above 500 °C has been observed.

Low melting temperature eutectic materials are ideal for heliostat applications^4^. Materials such as Lithium Nitrate (LiNO_3_), NaNO_3_ and KNO_3_ have eutectic temperature of 120 °C and below^5^. These eutectics and the result of other works^1^ show that the melting temperatures of quaternary salts with cations of Ca, K, Li and Na may be reduced to below 100 °C, allowing improved salt performance. In particular Nitrate anions can result in melting points of less than 80 °C, providing large amounts of improvement if the upper decomposition temperatures can remain stable^1^.

Metal Chloride salts have been introduced as a viable alternative for higher temperature applications chlorides, these have the advantages of a high latent heat and a high operating temperature (424–700 °C). Metal Chlorides do however have disadvantages including being corrosive and its melting point being considerably higher, reducing its overall usefulness to the concentrated solar power industry. For CSP applications an ideal salt would have a melting point lower than 142 °C and long term stability closer to that of the metal chloride mixtures. The advantages of Li additions have been seen to give lower melting temperatures for ternary mixtures providing potential cost savings when utilised as a transfer fluid^4,6^. Lower melting temperatures may allow an increased temperature differential, allowing a greater operating range and increasing the energy stored per kg^7^.

This work develops the combination of the commercial HITEC salt mixture with a variety of metal chlorides to create quaternary salts with the optimised properties for CSP.

Initial work into undisclosed chloride additions was undertaken by Peng *et al*., however whilst this work showed an improvement in salt operating temperatures it failed to state which additions were tested^2,8^. A wide variety of metal chlorides were tested within this study, with their melting points initially compared to a standard HITEC salt to ensure no increases in melting behaviour occur with salt additions. Chosen salts were then tested for their thermal decomposition. Data was obtained through use of Thermo-Gravimetric Analysis – Differential Scanning Calorimetry (TGA-DSC) techniques. The top performing quaternary mixtures were investigated further using long term stability tests w to prove overall lifespan.

## Results and Discussion

### Melting behavior of salts

**Figure 1:**
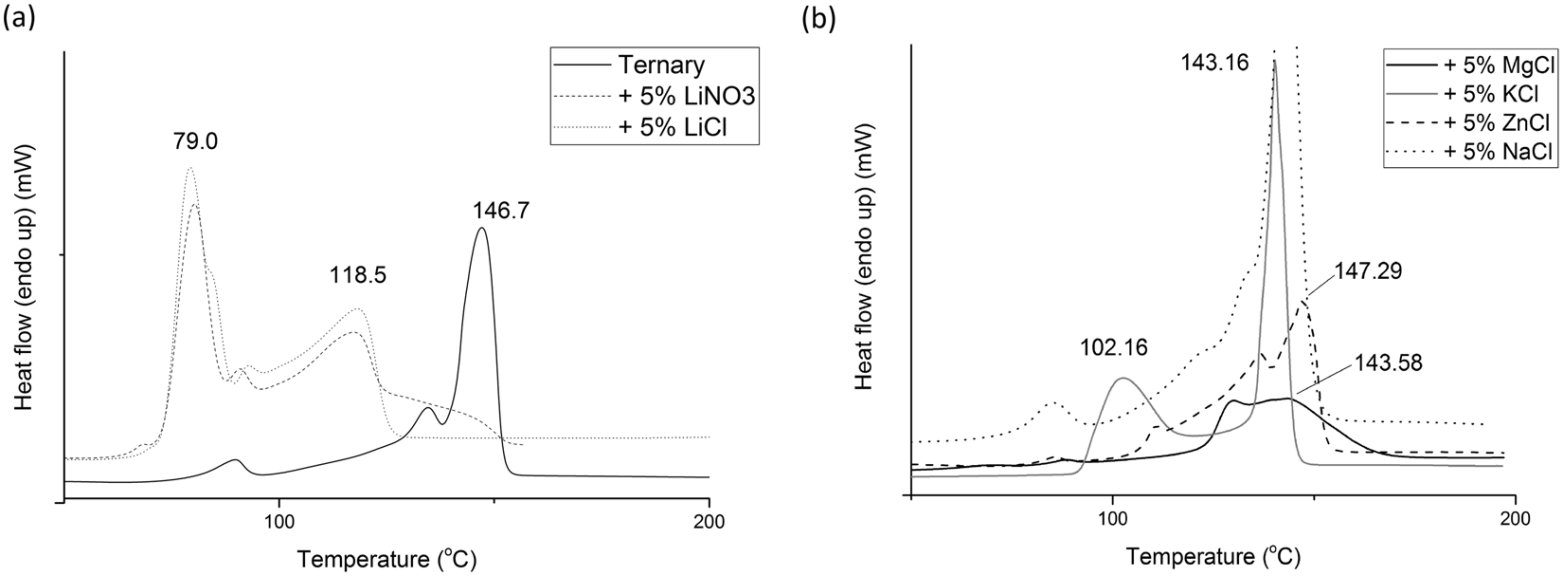
Melting behaviour of Ternary salts with varying chloride additions. Numbers indicate primary phase or state change temperatures.

Initially, a ternary salt of 53 wt% KNO_3_, 40 wt% NaNO_2_ and 7 wt% NaNO_3_ was tested using the DSC to serve as a control. This control showed a melting point of 146.7 °C, in agreement with other literature^9^. Following this a selection of salt additions of 5 wt% were prepared and tested in the DSC. Figure 1 shows the onset of melting for each mixture. It was seen that the additions of Magnesium Chloride (MgCl_2_) and Zinc Chloride (ZnCl_2_) both result in a reduction in the heat flow comparted to the ternary salt, as seen by the significant decrease in intensity. These MgCl_2_ and ZnCl_2_ also show no improvement in melting temperature, with the melting points being 143.58 and 147.29 °C respectively. Both have partial melting but the lower latent heat and lack of complete melting is undesirable. Sodium Chloride (NaCl) and Potassium Chloride (KCl) additions do not adjust the melting point considerably in comparison to the standard ternary salt, which allows these to be taken through for further study. LiCl was the most promising addition as the endotherm peak is significantly shifted down to 79 °C. This is an interesting addition of the Li salt. Whilst LiCl displays large amounts of deliquescence pre-treatments were undertaken in an attempt to minimise its impact on this study. These pre-treatments include keeping the salts in a low humidity environment, with heating remove any moisture prior to testing. For salt usage this may present a number of problems and salts would have to be kept moisture free prior to installation into the system, with care to prevent moisture ingress taken when in use. Pressure release valves may be needed to allow absorbed water to be removed at temperature without damaging the system. It can be seen that this is a direct result of the Li metal, as LiNO_3_ also has a similar drop. The melting point of pure LiCl is over 600 °C whilst LiNO_3_ is over 250 °C, so it can be assumed a new phase is being formed with the current eutectic. The molar ratios of these salts result in a significantly higher amount of Li cations being present in the LiCl mixtures. It is likely that as seen with the other salts Cl doesn’t cause the melting point to drop and in some cases raises it, this has a stabilising effect on the melting behaviour reducing the impact of the increased Li+ cation additions on the eutectic melt.

### Decomposition of salts

**Figure 2:**
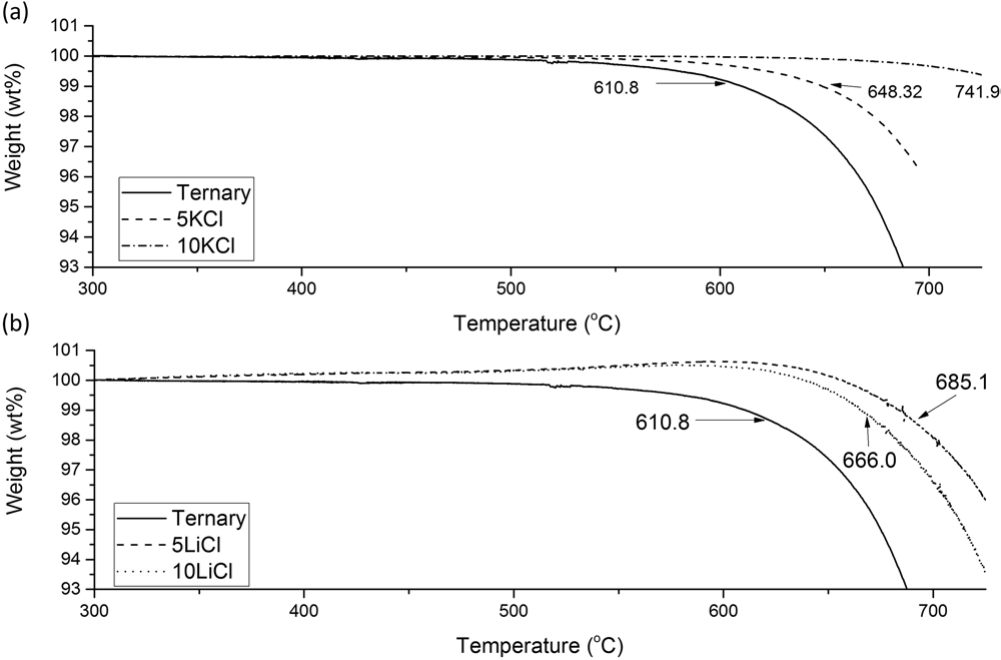
TGA analysis of the short term stability of (**a**) KCl; and (**b**) LiCl additions.

Short term behaviour of the chosen additions was tested using the TGA technique. Initial investigations showed metal-chlorides additions resulted in a stabilisation effect, as indicated by Peng *et al*.^2,9^. Onset of rapid decomposition was set at a weight of 99%, a 93% weight allowed the initial and rapid levels of degradation to be compared. Rapid decomposition was seen in the ternary control mixture at 610 °C as shown in Fig. 2(a). 5 wt% KCl additions resulted in a stability increase of 38 °C to 741 °C. Further additions of KCl result in further stability improvements. KCl has a large stabilisation effect allowing the operation at higher temperatures for periods of less than 30 minutes.

Lithium Chloride (LiCl) additions behave differently, with an increase in weight observed as shown in Fig. 2(b) suggesting oxidation. There is an initial stabilisation effect caused by the LiCl, allowing the sample to reach 685 °C before weight loss occurs. Additions of LiCl above 5 wt% result in a reduction in stability, this is shown by the lower temperature of decomposition seen in Fig. 2b. LiNO_3_ shows a reduced the decomposition temperature compared to many other nitrates^1^, it is believed that the Cl within the melt may aid in stabilisation, whilst the Li can cause some short-term instability decreases. Due to the melting behaviour similarities with LiNO_3_ and LiCl additions it is believed that the Li cation is separated from the Cl anion and reacts with the current nitrates in a similar way to that of the LiNO_3_ salt^10^. This implies a similar eutectic phase has been formed. Previous studies have shown that the LiNO_3_ phase is unstable within salt mixtures, degrading into Li_2_O_2_ and Li_2_O. These should exhibit peaks at 33° for Li_2_O_2_ and 34.8, 39 and 46.8° for Li_2_O. The Li_2_O_2_ peak is clearly identifiable in the LiCl salt, with some evidence of the Li_2_O peaks also being present, which possibly contribute to the structure changes, seen later in this work, with the larger eutectic phase. Similarly during the solidification, the Cl is located in the discrete lighter shaded phases. This Cl heavy phases solidifies first and will remain as a small amount of particulates in the melt, with the Li rich phase allowing earlier melting

### Long term stability of salts

**Figure 3:**
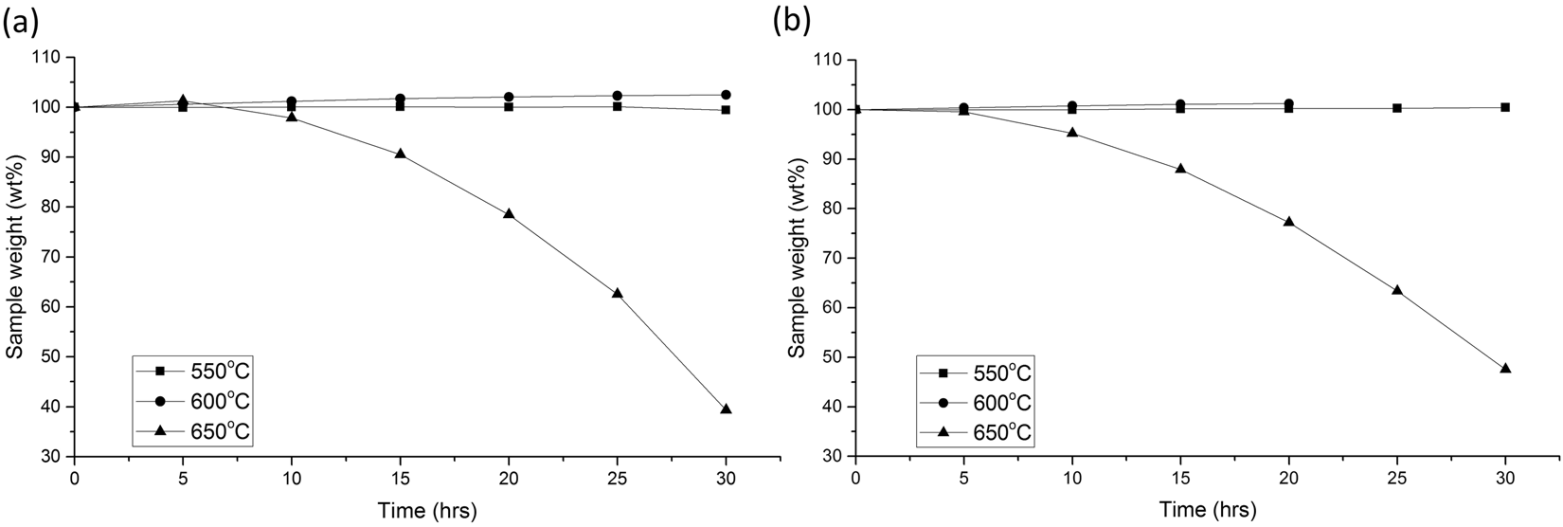
Long term decomposition of (**a**) ternary and (**b**) 5 wt%KCl addition salts.

**Figure 4:**
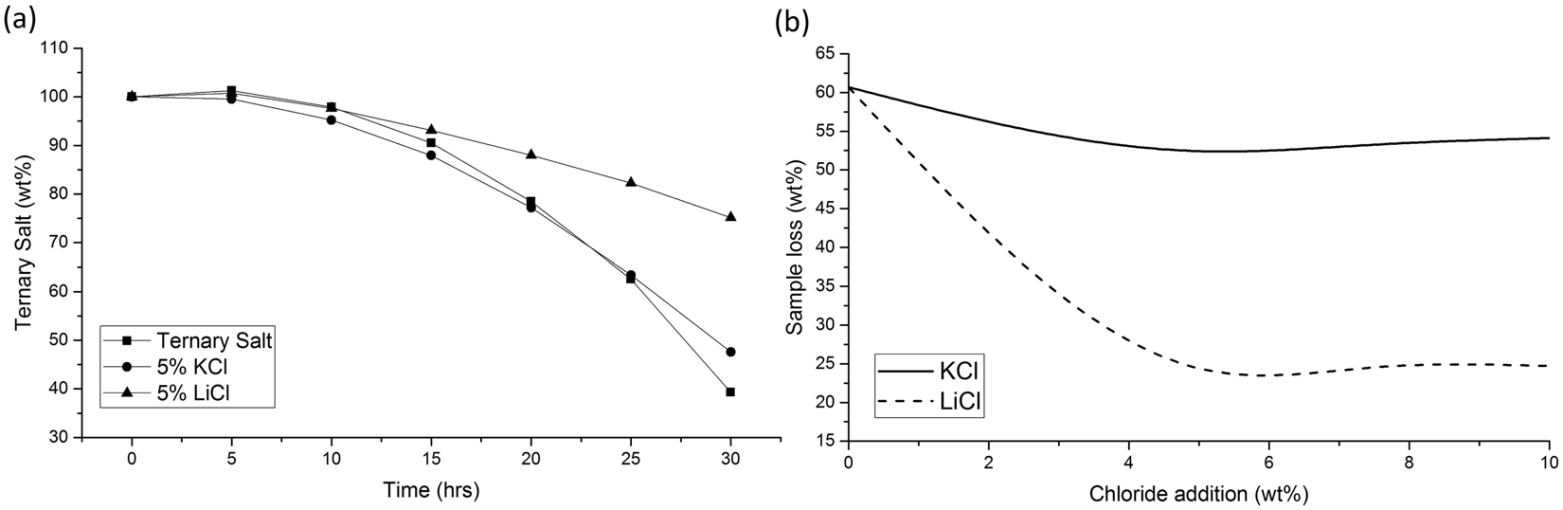
(**a**) Decomposition behaviour at 650 °C; (**b**) Salt weight loss after 30 hours at 650 °C.

Long term stabilities were tested using standard muffle furnaces over the 30-hour time span. Long term tests showed a noticeable variation in long term stability with additions of both KCl and LiCl. Any weight gains shown in Figs 3 and 4 can be attributed to oxidation of the salts. It can be seen in Fig. 3(b) that 5 wt% KCl does provide a small improvements, with no weight gain below 600 °C. The ternary salt, seen in Fig. 3(a), can be seen toshow a 5 wt% gain at 600 °C indicating some oxidation reaction. Figure 4(b) allows the coparison of varying chloride additions after 30 hours at 650 °C. Figure 4(a) shows the weight loss of salts at 650 °C. This shows that the weight loss was 60.1% for the ternary salt, whilst a 5 wt% addition of KCl reduces this weight loss to 52.4 wt%. Weight loss of the all salts was low at 550 and 600 °C, possibly due to some cooling in the furnace. Small weight gains are seen in the ternary salt at all temperatures, most probably due to the conversion between NO_2_ to NO_3_. The most noticeable improvement is with the addition of LiCl, where a 5 wt% addition results in a weight loss of only 24.84 wt%. Figure 4(b) also shows that there is little or no improvement with additions of higher than 5 wt% LiCl additions. This ties up the data from the short-term experimentation.

### XRD analysis

**Figure 5:**
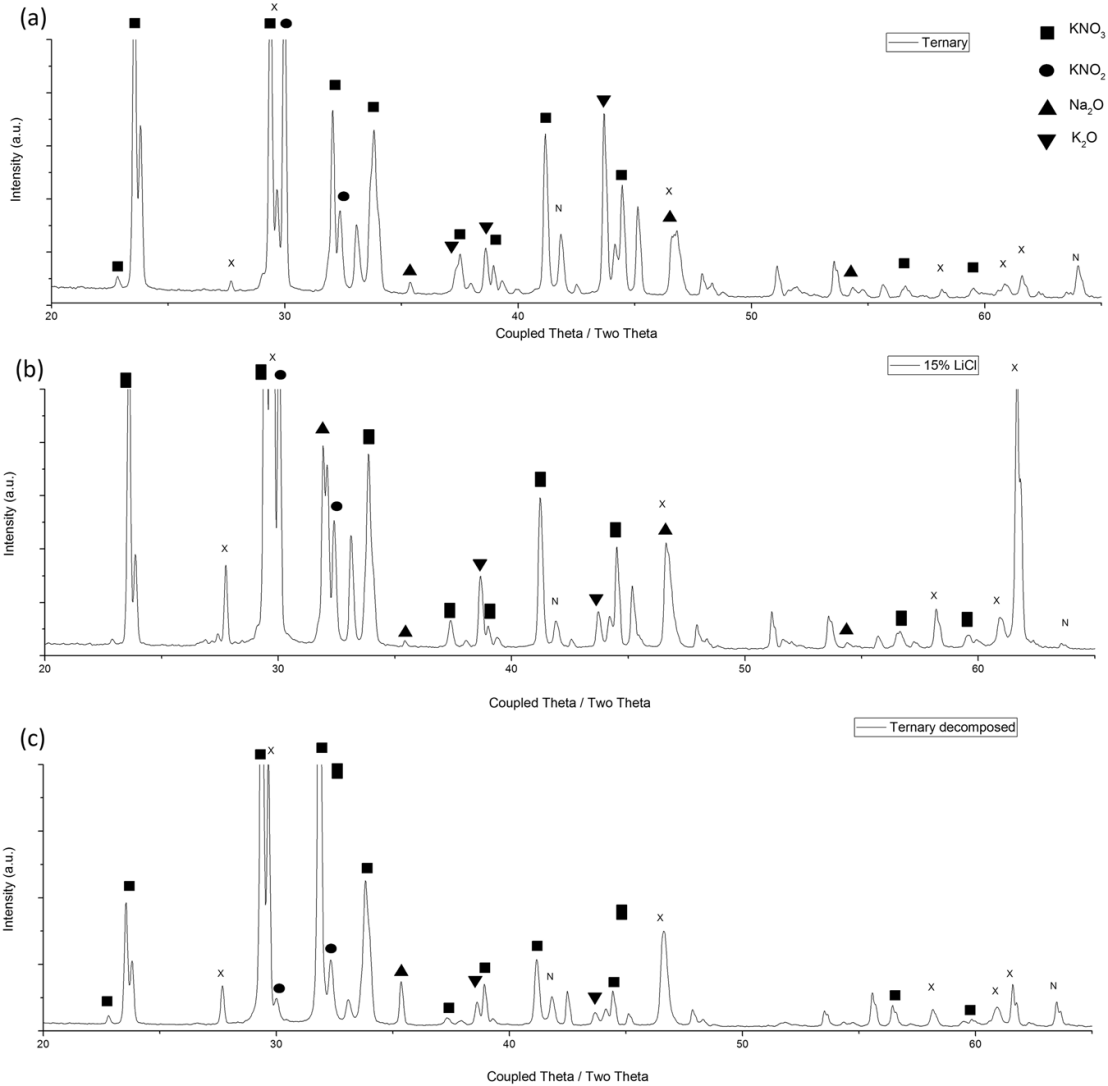
XRD trace of (**a**) ternary salt; (**b**) 5 wt% LiCl addition; and (**c**) ternary salt after 30 hours at 650 °C.

In order to determine the phases present, pure salts were initially scanned and used to identify the peaks of pure salt, other phases were sourced from an open database. Figure 5 shows the effect of the LiCl additions on the solid crystal structure of the ternary salts. There are 6 noticeable increases in peaks and two significantly reduced peaks with the addition of LiCl, marked by x and n respectively in Fig. 5. These peak increases are seen in both the LiCl containing samples and decomposed ternary samples (Fig. 5b and c). It is likely the LiCl allows for a room temperature transformation to produce a phase that shares a similar crystal structure to that of the decomposed ternary phase. The most likely decomposition phase, Sodium oxide (Na_2_O), would increase the melting point. This is seen to form in the LiCl samples, however it doesn’t raise the melting point of the eutectic, so it may be an additional phase. Given the change in melting point it can be assumed that the peaks, marked x in Fig. 5, (27.72, 29.7, 46.6, 58.2, 60.9 and 61.7) correspond to a beneficial phase that enables the more rapid melting.

Following the resultant decomposition of the ternary phase, there is a reduction in the primary KNO_2_ peak and an increase in the peak relating to KNO_3_. This is indicative of an oxidation reaction occurring. There is a noticeable increase in the Na_2_O peaks, whilst a few peaks relating to KNO_2_ and K_2_O decrease, indicating that these are the primary phases decomposing. A similar peak (at 27.72°) can be seen in the XRD trace obtained in the case of HITEC with LiCl additions to that observed in the case of the decomposition phase. An increase in the peaks relating to Na_2_O are also present in both curves. Na_2_O is a high temperature phase that should remain solid until 1132 °C, however no signs of any solid are present at elevated temperatures, indicating that it is dissolving into the melt.

Resultant decomposition phases for ternary and LiCl containing phases are remarkably close to each other, indicating that the same bulk decomposition is occurring through the same process. There is less weight loss with the LiCl stabilising addition, possibly due to binding of Na and O_2_ preventing further oxidation and decomposition. There is a new decomposition phase forming with the LiCl indicated by the increase in peaks, particularly at 22°.

### SEM imaging

**Figure 6:**
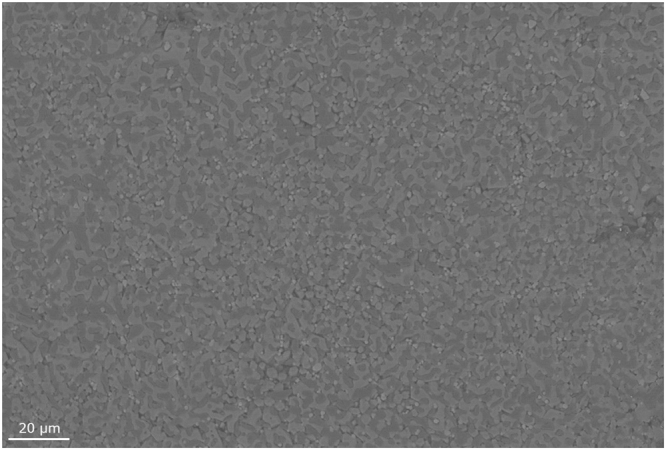
SEM image of Ternary Salt.

**Figure 7:**
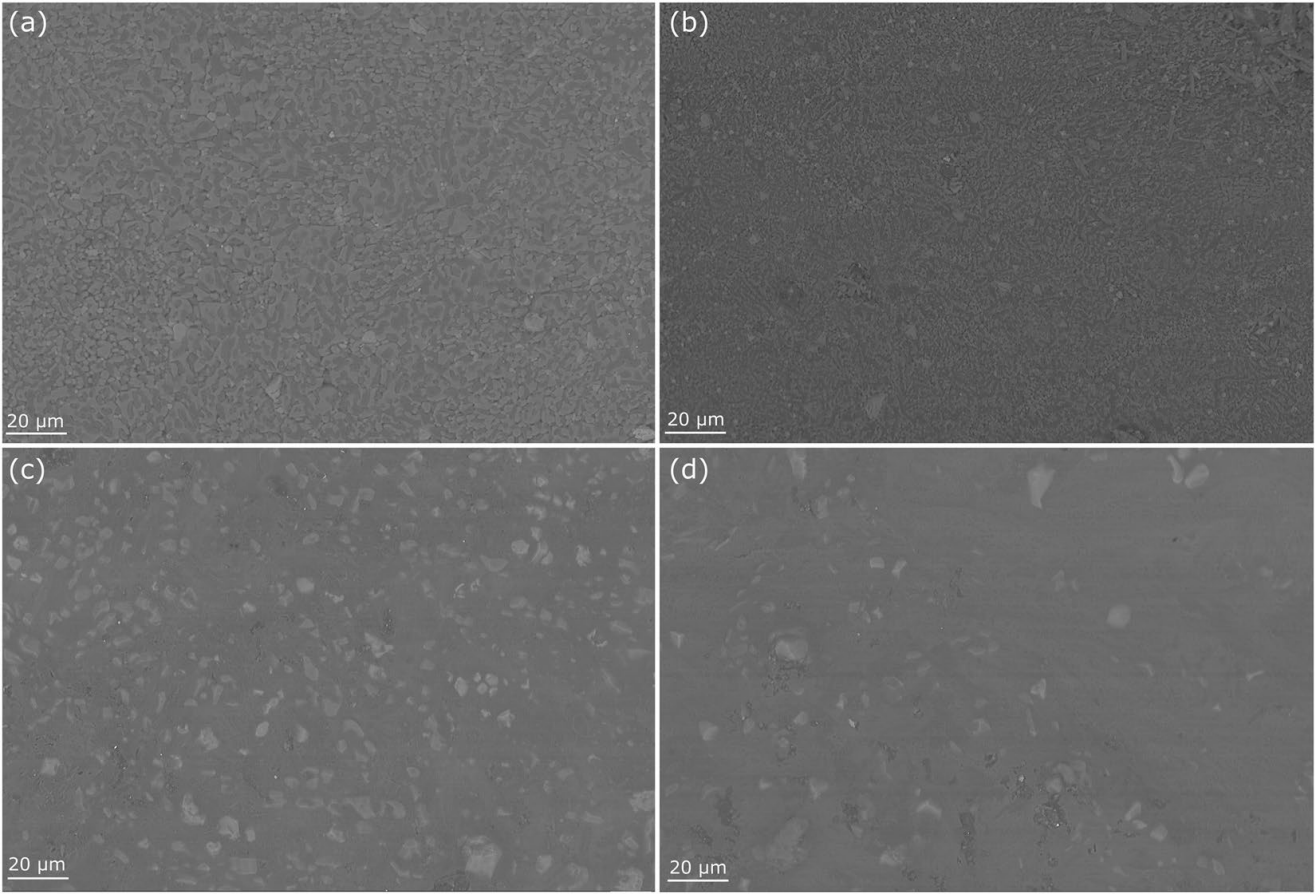
SEM images of Chloride additions (**a**) 10% KCl, (**b**) 20% KCl, (**c**) 10% LiCl and (**d**) 20% LiCl.

In addition to attempting to determine phases with XRD, SEM imaging allowed the viewing of the solidified structure of the material. A standard ternary salt, shown in Fig. 6, has a fine structure with 3 separate phases possible to identify using Back Scatter Electrons. With the addition of KCl (see Fig. 7a and b), there is a slight increase in one phase, most likely a high potassium phase like K_2_O. This changes the morphology of the salts makeup giving larger dendritic like structures. However due to the penetration depth of the electrons when using EDS analysis, it is difficult to scan phases without viewing deeper into the sample resulting in a mixed reading. Figure 7 show higher salt additions than that discussed in the prior data. Whilst the structural changes are still detectable at lower addition levels, by providing an overdose of salt within the salt we are able to exaggerate the microstructure to make it more visible.

Lithium additions (Fig. 7(c) and (d)) are seen to have a much greater effect on the solid structure of the salts. Although due to the limitations of the EDS it is impossible to detect Li within a phase, the structural changes are noticeable, with considerable increases in the darkest matrix phase. This can be related to the increase in Li present, relating nicely to the generation of additional phases detected on the XRD. There are slight concentration variations within the darker matrix, seen in the Fig. 7d as shade variations. These are difficult to identify in the EDS software, as Li, which is a likely constitute of these phases, cannot be detected.

## Conclusions

In comparison to the standard ternary HITEC salt, the additions of metal chlorides showed an overall improvement in the long-term stability of the salts. Additions of KCl and LiCl are shown to not raise the eutectic melting point of the salts, ensuring no detrimental effects on salt performance. The addition of 5 wt% LiCl allowed partial melting prior to the 140 °C eutectic point, further increasing the operating range of the material within solar-thermal generation and thermal storage applications.

Analysis indicates a new phase has been formed with the LiCl additions, which at room temperature allows more rapid melting. Small additions of LiCl reduce the weight loss over long time periods from 61% to 25% in comparison to standard ternary salts. Given LiNO_3_ additions did not result in the stability increase seen with LiCl, it can be deduced that additional Nitrates do not improve stability and that Cl is the initial stabilising agent. With the Li in this case, allowing a phase with a lower melting temperature to be formed. Overall, small LiCl additions can potentially allow the standard HITEC salt to be molten from 100 °C, then reach over 630 °C in the short term, with the potential to operate longer term at temperatures above 550 °C, with no signs of oxidation. This provides a potential working range improvement of over 100 °C in comparison to the standard HITEC salt.

## Materials and Methods

### Materials and synthesis

Quaternary eutectic salts were prepared by mixing individual component salts, with analytical reagent grade materials being sourced from Sigma-Aldrich, and kept in a vacuum to dry prior to weighing and mixing. The preparation is explained in detail in other works^2,9^. The HITEC mixture (53 wt% KNO_3_ – 40 wt% NaNO_2_ – 7 wt% NaNO_3_) was prepared initially, with additions of chlorides added later (KCl, LiCl, CaCl, ZnCl, NaCl and MgCl). After mixing, salts were heated to 200 °C for 2 hours to allow melting and homogeneity to be reached ready for testing.

### Experimental procedure

Differential Scanning Calorimetry (Perkin Elmer – DSC 4000) was used to generate the actual eutectic melting point of the mixtures. With this method, 10–20 mg of sample were put in to an Aluminium pan, alongside an empty reference pan. Samples were heated at a standard rate of 20 °C/min, to 300 °C, in a nitrogen atmosphere with a 20 mL/min flow rate^11^. This cycle was completed twice, to ensure any moisture absorbed was is removed in the first run.

Following this, a Simultaneous Thermal Analysis (Perkin Elmer – STA 6000) was used to examine the short-term decomposition behaviour. 1–20 mg samples were placed into an Alumina crucible and heated at a slower rate of 10 °C/minute in air to 700 °C. For long term decomposition, 10 g of salt were placed in porcelain crucibles for periods of up to 30 hours, where they were heated to 550, 600 and 650 °C. Ramp rates of 3.5 °C/min were used to ensure the crucibles did not fracture and weight measurements were taken every 5 hours. Due to the ability of LiCl to absorb water all salts were mixed in a low humidity environment to reduce any moisture absorption.

Powder analysis X-Ray Diffraction (XRD) was undertaken on a D8 Discover (Bruker), with a Cu source (40 kV, 40 mA). Scans were taken at 20–60 2θ over a period of 20 min. The phase matching was completed using the Crystallographic Online Database^12^.

Scanning Electron Microscopy (SEM) was undertaken using a Zeiss Evo SEM, using a tungsten source set to 10 kV with a probe current of 250pA. A oxford instruments backscatter electron (BSE) detector was used to generate an image with contrast for each of the phases at 500 × zoom. Prior to weight loss and SEM preparation all samples were preheated and held at 160 °C and held for an hour. This allowed any retained water to be removed ready for testing.
